# Exploring relationships over time between psychological distress, perceived stress, life events and immature defense style on disordered eating pathology

**DOI:** 10.1186/2050-7283-1-27

**Published:** 2013-12-05

**Authors:** Phillipa Hay, Sarah Elizabeth Williams

**Affiliations:** School of Medicine, University of Western Sydney, Sydney, Australia; School of Medicine, James Cook University, Townsville, Australia

**Keywords:** Psychological distress, Perceived stress, Life events, Defense style, Eating disorders

## Abstract

**Background:**

Perceived stress, immature defense style, depression and anxiety and negative life events all are known to be associated with eating disorders. The present study aimed to investigate the relationships between these factors and their relative strength of association with eating disorder symptoms over time.

**Methods:**

This research was embedded in a longitudinal study of adult women with varying levels of eating disorder symptoms and who were initially recruited from tertiary educational institutions in two Australian states. Four years from initial recruitment, 371 participants completed the Eating Disorder Examination- Questionnaire (EDE-Q) for eating disorder symptoms.

Kessler-10 Psychological Distress Scale (K-10) as a measure of depression and anxiety, a Life Events Checklist as a measure of previous exposure to potentially traumatic events, the Defense Style Questionnaire (DSQ) and the Perceived Stress Scale (PSS) to determine perceived stress. One year later, in year 5, 295 (878.7%) completed follow-up assessments including the EDE-Q. The questionnaires were completed online or returned via reply paid post.

**Results:**

All four independent factors were found to correlate significantly with the global EDE-Q score in cross-sectional analyses (all Spearman rho (r_s_) >0.18, p < 0.01) and at one year follow-up (all r_s_ > 0.15, all p < 0.05). In multivariate linear regression modeling adjusted for age and year 4 global EDE-Q scores, perceived stress and psychological distress scores were significantly associated with year 5 global EDE-Q scores (p = 0.046 and <0.001 respectively).

**Conclusions:**

Psychological distress, and to a lesser degree perceived stress had the strongest association with eating disorder symptoms over time The findings support further investigation of interventions to reduce distress and perceived stress in adult females with disordered eating.

## Background

Three main eating disorders are defined in the DSM-5 (American Psychiatric Association 2013): anorexia nervosa (AN) which is defined as a refusal to maintain body weight at or above minimum normal weight for age and height, bulimia nervosa (BN) which is delineated as recurrent episodes of binge eating followed by regular compensatory behaviours, and binge eating disorder (BED) which is delineated as recurrent binge eating without compensatory behaviours. Eating disorders are a pertinent public health issue in North America and elsewhere due to their prevalence and their association with other psychopathology, role impairment, and history of being under-treated (Hudson et al. 2007). Psychological and social features such as mood intolerance or “an inability to cope appropriately with certain emotional states” are known to contribute to the onset and/or maintenance of eating disorder symptoms (Fairburn et al. 2003). This present paper explores the relationships between four such psychosocial factors, namely psychological distress from affective symptoms, defense style, perceived stress and life events, and eating disorder symptoms. In this background we present research reporting the association between these four features and eating disorder symptoms.

Affective symptoms, the first factor under consideration, are a common co-morbidity of eating disorders (Swinbourne and Touyz 2007; Arajo et al. 2010; Greeno and Wing 1994; Spoor et al. 2007; Kaye et al. 2004). Many studies investigating the relationship between eating disorders and depression or anxiety are cross sectional, and thus conclusions regarding causal relationships are unable to be made. Nonetheless as in Fennig and Hadas (2010), depression has been shown to amplify eating disorder severity. We have also found that a general measure of affective symptoms or psychological distress was more strongly associated with weight stability than eating disorder symptoms in a longitudinal study of women with disordered eating (Darby et al. 2009).

Coping strategies are thoughts and behaviors practiced in response to negative or stressful life events to manage and tolerate internal or external demands (Lazarus and Folkman 1984; Endler et al. 1993). In many, but not all (Paxton and Diggens 1997) studies, women with eating disorders have been found to be more likely to employ less effective coping mechanisms than women without eating disorders (Troop et al. 2008; Freeman and Gil 2004; VanBoven and Espelage 2006; Sulkowski et al. 2011; Garcia-Grau et al. 2001). Such maladaptive coping styles can result from an immature or less well developed defense style. Blaase and Elklit (2001), reported that woman currently suffering from an eating disorder use significantly more immature defenses than women without such a disorder. This has been confirmed by most other studies including Stein et al. (2003) with the exception of Sullivan et al. (1994). Furthermore, we have found that an immature defense style was associated with poorer mental health related quality of life at 2-year follow-up in a longitudinal community study of women with disordered eating (Hay et al. 2010) although psychological distress had a stronger association. We propose that this may have been because employing less adaptive defense mechanisms leads to experiencing greater psychological distress in response to stressful events (Endler et al. 1993).

A high frequency of stressful life events preceding the onset of an eating disorder has been reported (e.g., Schmidt et al. 1992; Raffi et al. 2000; Welch et al. 1997). Numerous studies have also shown that women suffering from eating disorders are generally exposed to more life events than the general population (Sharpe et al. 1997; Schmidt et al. [Bibr CR48][Bibr CR45][Bibr CR47][Bibr CR46]; Blaase and Elklit 2001; Lacey et al. 1986; Pike et al. 2006; Welch et al. 1997; Strober 1984). The findings of Grilo et al. (2012) suggest that the occurrence of negative stressful life events, most notably higher work stress and higher social stress, represent significant warning signs for relapse among women in remission from BN and other eating disorders.

In contrast to actual life events, which may be variably stressful to an individual, the construct of perceived psychological stress measures the degree to which one perceives aspects of one’s day to day life as unpredictable, uncontrollable or overloading (Cohen et al., 1983). Inconsistent findings have however been found in the relationship between perceived psychological stress and disordered eating. Several studies have reported significant relationship exists between perceived stress and disordered eating (Ball and Lee 2000; Groesz et al. 2012; Blaase and Elklit 2001; Wolff et al. 2000; Beukes et al. 2010; Pendleton et al. 2001. However, Ball and Lee (1999) demonstrated that high psychological distress but not perceived stress was significantly correlated with eating disorder symptoms levels. Furthermore, perceived stress did not predict eating disorder symptoms over a 6-month follow-up according to Ball and Lee (1999).

The relationships between perceived stress, depression and anxiety or general psychological distress, defense style, experiencing negative life events and eating disorder symptoms in young women are thus complex and incompletely understood. Despite the likelihood that these are correlated with each other as well as with eating disorder symptoms, to our knowledge, no previous study has looked at independent effects of these particular variables together in a single analysis. In Rojo et al. (2006), stress, in particular chronic and severe stress was found to be associated with the development of eating disorders when mediated by the presence of psychiatric co morbidities, which were depressive and anxiety disorders. The results indicated that though stress preceded 25% of eating disorder cases, psychiatric co-morbidity in the absence of stress preceded 31% of cases. Similarly, a study on disordered eating in Young Chinese Women (Chen et al. 2012) showed that though there was no significant direct effect of perceived stress to disordered eating, negative affect (depression and anxiety) significantly mediated the relationship between perceived stress and disordered eating. The present study was thus designed to further investigate the relationships between perceived stress, psychological distress as well as negative life events and immature defense style with disordered eating in a large longitudinal cohort of adult women, namely those at most risk of an eating disorder (Hudson et al. 2007).

We hypothesized that higher levels of perceived stress, higher levels of psychological distress, an immature defense style and more frequent life events will each have a strong association with eating disorder symptoms. Furthermore, the effects of psychological distress would have the strongest independent association with eating disorder symptoms over time.

## Methods

### Participants

**Figure 1:**
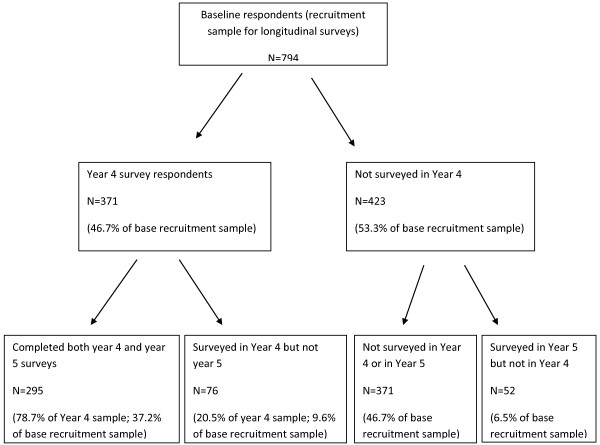
**Participant flow.**

Participants of the present study were recruited four years prior to the present study using advertisements placed across four institutions of tertiary education in Queensland and Victoria. The study did not specifically recruit for women who were having trouble with eating/body image but rather for people interested in participating in a “Women’s health and wellbeing survey”. Those who were approached via email were given the option to do the questionnaire online while other participants were approached by various means including bulletins and halls of residence and directly, and were given the questionnaire in hard copy with reply-paid envelopes. Due to these methods of recruitment, it was not possible to measure the overall response rate to the recruitment survey or to investigate the characteristics of non-respondents. To date, 6 waves of assessment over 9 years in total have been conducted. The present study sample (see Figure 1) was composed of the 371 participants (of an initial 794 respondents) who completed the four year survey and the 295 (78.7% response) who completed both the year four and the year five surveys. The participants in the present sample were an average of two years older (p < 0.05) with higher levels of eating disorder symptoms (but not general psychological distress) compared to the 423 who were not included from the initial group of 794 women (global EDE-Q scores of 1.9 SD 1.3 versus 1.7 SD 1.3, p < 0.05). Features of those in the initial sample with clinical levels of eating disorder symptoms have been described previously (Hay et al. 2012). At baseline, 221 were described as ‘symptomatic’ i.e. they had had current extreme weight/shape concerns and/or current regular (e.g. occurring weekly over the past three months) binge eating and/or any extreme weight control behaviours such as self-induced vomiting and/or laxative/diuretic use and/or fasting or severe food restriction and/or ‘driven’ exercise withpredominately of binge eating disorder or a similar type of eating disorder.

The study was approved by the human research ethics committees (HREC) of the universities involved and University of Western Sydney as lead HREC (Approval number 07/240). All participants completed written informed consent and there were no children requiring consent from a parent or guardian.

### Assessment instruments

#### Eating Disorder Examination Questionnaire (EDE-Q)

The EDE-Q is a 36-item self-report questionnaire focusing on the previous 28 days (Fairburn and Beglin 1994; Wilfley et al., 1997). The EDE-Q has been validated in community and clinic samples of people with eating disorders. A global score of eating disorder attitudes and restraint, and four sub-scales (i.e. shape, weight and eating concern and dietary restraint) can be derived and it assesses frequency of specific diagnostic behaviors such as binge eating and driven exercise. Mond et al. (2006) have reported Australian norms. The four subscales have been found to have good reliability (alpha and test-retest reliability coefficients ≥ 0.8) and moderate predictive validity in identifying probable cases of the more commonly occurring eating disorders (Se = 0.8, Sp = 0.8, PPV = 0.5) and the measure appeared well suited for use in prospective epidemiological studies (Mond et al., 2004).

#### Kessler 10 psychological distress scale (K-10)

The K-10 is a 10-item questionnaire measuring 10 symptoms of mental health oriented to depression and anxiety (Kessler et al. 2002). With the aim to measure the level of distress and severity associated with psychological symptoms in population surveys, the K-10 is extensively used internationally, including in the WHO World Mental Heath Survey and by government organizations in Australia, Spain, Colombia and Peru (Terrez et al. 2011). The advantages of the K-10 are its brief nature (10 questions with 2–3 minute completion time), its broad screening ability, its strong psychometric properties (Kessler et al. 2002; Donker et al. 2010) and its ability to discriminate DSM-IV disorders from non-cases (Kessler et al. 2002). It focuses on the previous 28 days to the questionnaire and each question can be answered from 1–5 in an ordinal scale, 1 being “none of the time” and 5 being “all of the time”. Scores range from 10–50 with a higher score indicative of more distress and a score 16 or more indicative of risk of mental illness (Andrews and Slade 2001).

#### Life events checklist

The 37-item Life Events Checklist is a measure of previous exposure to health, perinatal, traumatic, family and interpersonal, socio-economic and/or legal life events. It was originally developed by the National Centre for Post Traumatic Stress Disorder to diagnose subjects suffering from Post Traumatic Stress Disorder. In an evaluation of the Life Events Checklist, its performance in both the clinical and non-clinical samples was concluded to be encouraging (Gray et al. 2004). It is a 37 item simple yes/no self report questionnaire, indicating if the participant has experienced a variety of life events over the last 12 months (Dobson et al. 2005)). It was developed for use in the Australian Longitudinal Study on Women’s Health (ALSWH), where norms were established (Women’s Health Australia 1997). It is scored by summing the life events in each domain of health, perinatal, trauma, family or other interpersonal, socioeconomic or legal events and to provide a total number.

#### Defense style questionnaire - 40 item (DSQ-40)

Defense mechanisms are coping strategies exercised to protect the individual from anxiety and excessive negative affect to maintain self esteem (Zeigler-Hill and Pratt 2007). However, unlike mature defenses, neurotic and immature defenses are thought to fulfill this role at the expense of interpersonal relationships and a sense of reality. Bond et al. (1983) developed the Defense Style Questionnaire (DSQ) with the rationale of the hierarchy of defense styles from mature via neurotic to immature defense styles. The DSQ-40 is comprised of 40 items, which are given a rating by the subject from 1 (strongly disagree) to 9 (strongly agree). 20 defense mechanisms are tested for with 2 items for each defense. The 3 specific defense styles are mature, neurotic and immature and the various mechanisms are organized within them. It is scored by summing and dividing by two the 2 items for each defense mechanism. The three defense styles are scored by summing the scores for each mechanism within the style and dividing by the number of defenses for that style. The mature defense styles include the mechanisms of humor, suppression, sublimation, and anticipation. The neurotic style consists of reaction formation, idealization, pseudo-altruism, and undoing. The immature defense style mechanisms tested for are rationalization, autistic fantasy (e.g., “I get more satisfaction from my fantasies than from my real life”), displacement, isolation, dissociation, devaluation, splitting, denial, passive aggression, summarization, acting out, projection (e.g., “I am sure I get a raw deal from life”; Zeigler-Hill and Pratt 2007). The results of the DSQ therefore discriminate among different styles of pathological coping and are viable in a non-clinical as well as clinical setting (Sammallahti et al. 1996). The DSQ has good reliability, internal consistency, temporal stability and moderate validity (Andrews et al. 1993 Sammallahti et al. 1996). A higher score is indicative of a higher level of presence of the defense style. Andrews et al. (1993) have reported the following Australian community norms in 338 participants: Immature mean 3.5 (SD 0.95); Neurotic mean 4.3 (SD 1.28); Mature mean 5.8 (SD 1.15).

#### Perceived Stress Scale (PSS)

Stress is the perceived or actual threat on physical and/or psychological homeostasis of the human body (Chrousos 1998). The PSS was developed by Cohen et al. (1983) to meet the need of an assessment of perceived stress, which could be administered without such limited conditions to specific groups. The PSS is a self-report questionnaire with the aim to find the degree to which situations in the subject’s life are perceived as stressful and specifically the degree to which one perceives aspects of one’s day to day life as uncontrollable, unpredictable and over loading (Cohen et al. 1983). Though originally a 14-item scale, the 10-item version showed stronger psychometric characteristics (Cohen and Williamson 1988). 10 questions are asked to find the frequency of specific feelings and thoughts during the last month, with the subject able to respond from 0 = never to 4 = very often (Cohen et al. 1983). Scores may range from 0 to 40, with higher composite scores indicative of greater perceived stress. The advantages of the PSS, which has made it so popular, is its robust psychometric qualities and concise length (Reis et al. 2010).

### Statistical analyses

Data were inspected for normality. The Spearman ranked correlations test (Spearman rho (r_s)_) was used because of non-normality of some data. Multivariate linear regression analyses were conducted to determine the strength of association of perceived stress, psychological distress, life event number in preceding year and level of immature defense style (independent variables) on concurrent (year 4) global EDE-Q scores adjusting for age and 12-month (year 5) global EDE-Q scores (dependant variables) adjusting for year 4 EDE-Q scores and age. A significance level of < 0.05 was employed for all tests. Analyses were conducted using the SPSS for Windows version 20.

## Results

### Demographics

Of the 371 participants (46.7% of first year respondents) who completed the four year follow up survey, 19.1% were currently studying, 68.5% were employed, 49.3% were married or living as married, 34.5% had children, the highest level of education of majority of respondents (55.1%) was a bachelor’s degree and the majority lived with a partner/husband (49.7%). Other features of the sample are found in Table 1.

**Table 1:** **Descriptive data of present study participants**

	Response number (n)	Mean	Std deviation	Median	IQ range
**Age**	368	32.9	11.5	27.0	24.0-39.8
**BMI (kg/m** ^**2**^ **)**	356	25.5	6.1	24.3	21.3-27.9
**Perceive Stress Scale**	360	15.7	7.1	15.0	11.0-20.0
**Defense Style Questionnaire**					
Mature	352	5.4	1.1	5.4	4.6-6.3
Neurotic	362	4.4	1.1	4.4	3.8-5.1
Immature	348	3.4	0.9	3.4	2.8-3.9
**Eating Disorder Examination- Questionnaire**					
Weight concern subscale	366	2.0	1.5	1.9	0.6-3.0
Eating concern subscale	359	0.9	1.1	0.4	0.2-1.2
Shape concern subscale	358	2.3	1.5	2.1	1.0-3.5
Restraint subscale	365	1.5	1.3	1.0	0.4-2.4
Global Score	348	1.7	1.2	1.4	0.6-2.4
**Kessler 10 Psychological Distress Scale**	362	17.3	6.4	16	13.0-20.0
**Life events Checklist**					
Health life event	364	0.2	0.5	0.0	0.0-0.0
Perinatal life event	364	0.0	0.2	0.0	0.0-0.0
Trauma life event	365	0.4	0.7	0.0	0.0-1.0
Family personal life event	363	1.2	1.3	1.0	0.0-2.0
Socioeconomic life event	361	1.8	1.3	2.0	1.0-2.0
Legal life event	365	0.1	0.3	0.0	0.0-0.0
Total Life Events Score	348	3.8	2.4	3.0	2.0-5.0

Two hundred and ninety-five individuals completed both the year four and fifth year survey. Twenty percent of these were currently studying, 66.5% were employed, 51.6% were married or living as married, 33.2% had children. The highest level of education of majority of respondents was a bachelor level degree (55.3%), and the majority lived with a partner or husband (52.2%).

### Analysis

Number of life events (r_s_ = 0.18), levels of perceived stress (r_s_ = 0.33), psychological distress (r_s_ =0.37) and immature defense style (r_s_ = 0.23) all correlated positively with global EDE-Q scores in the concurrent year (p ≤ 0.001) and with each other (see Table 2) with the exception of life event number and level of immature defense style. Number of life events (r_s_ = 0.15), levels of perceived stress (r_s_ = 0.36), psychological distress (r_s_ =0.40) and immature defense style (r_s_ = 0.25) all also correlated positively with global EDE-Q 12-months later (p ≤ 0.05) (Table 2). In separate linear regression models, all four independent variables were significant predictors of initial global EDE-Q scores (Models 1–4) and at year 5 follow-up only psychological distress and perceived stress were significantly associated with global EDE-Q scores (Table 3).

**Table 2:** **Correlations (Spearman’s rho (r**_**s**_ **)) of dependent variables with year 4 and year 5 global eating disorder examination questionnaire scores**

	Yr 4 EDE-Q global	Yr 4 Perceived Stress Scale	Yr 4 DSQ Immature	Yr 4 K-10	Yr4 Total Life Events
**Yr 4 EDE-Q global**					
r_s_	1				
N	348				
**Yr 4 PSS**					
r_s_	0.334***	1			
N	337	360			
**Yr 4 DSQ immature**					
r_s_	0.232***	0.418***	1		
N	328	339	348		
**Yr 4 K-10**					
r_s_	0.372***	0.710***	0.455***	1	
N	344	352	342	362	
**Yr 4 Total Life Events (n)**					
r_s_	0.184**	0.358***	0.107	0.375***	1
N	325	338	326	339	348
**Yr 5 EDE-Q global**					
r_s_	0.745***	0.363***	0.245***	0.403***	0.151*
	278	283	277	287	277

EDE-Q = Eating Disorder Examination- Questionnaire, K-10 = Kessler-10 Psychological Distress Scale, DSQ = Defense Style Questionnaire, PSS = Perceived Stress Scale, *p < 0.05; **p < 0.01; ***p < 0.001.

**Table 3:** **Multivariate linear regression analyses of dependent variables with year 4 adjusted for age and year 5 EDE-Q scores adjusted for age and year 4 global EDE-Q scores**

	Dependent variables	Independent variables	F(df)	Adjusted R^2^	p
**Model 1**	Global EDE-Q year 4	Level of immaturity	20.2,1,325	0.061	<0.001
	Global EDE-Q year 5	Level of immaturity	2.4,1,263	0.554	<0.122
**Model 2**	Global EDE-Q year 4	Preceding life events	14.4,1,323	0.050	<0.001
	Global EDE-Q year 5	Preceding life events	0.06,1,261	0.548	0.814
**Model 3**	Global EDE-Q year 4	Psychological distress	82.64,1,341	0.200	<0.001
	Global EDE-Q year 5	Psychological distress	12.5,1,274	0.571	<0.001
**Model 4**	Global EDE-Q year 4	Perceived stress scale	58.6,1,334	0.17	<0.001
	Global EDE-Q year 5	Perceived stress scale	4.02,1,267	0.552	<0.046

EDE-Q = Eating Disorder Examination-Questionnaire.

## Discussion

This present study investigated the relationships between level of psychological distress, immaturity of defense style, perceived psychological stress, number of preceding life events and eating disorder symptoms in a sample of adult women recruited four years previously from institutions of tertiary education education in Australia. The findings supported the hypothesis that psychological distress would have the strongest independent association on eating disorder symptoms over time although perceived stress also was significant. The findings also partly support those of Chen et al. (2012) who in a cross-sectional study reported that levels of depression and anxiety mediated the effects of perceived stress on disordered eating in young Chinese women. The findings differ from Ball and Lee (2002) who found that that perceived stress did not predict eating disorder symptoms over time when controlling for eating disorder symptoms at baseline. It could be argued that perceived stress is a ‘proxy’ variable for psychological distress, or indeed both are measuring a closely similar construct, as they were very highly correlated (Kraemer et al. 2001).

The findings that immature defense style correlated significantly with global eating disorder scores in both the concurrent year and at 12-months supports the findings of Stein et al. (2003) which suggested that combined use of immature and neurotic defenses may be associated with a greater risk to develop a partial eating disorder. Furthermore the correlation found between the number of life events and EDE-Q global score in both the concurrent year and at 12-month follow up accords with findings of most previous studies including Raffi et al. (2000), Pike et al. (2006) and Grilo et al. (2012). However, neither of these two factors were significantly associated with eating disorder symptoms over the year follow-up when controlling for preceding eating disorder symptoms.

The strengths of this study include the large sample size (n = 371) and 78.7% response rate of individuals followed over both years of the study, and the longitudinal design and the use of validated instruments supports the integrity of this study’s findings. Two important limitations of this study are that the participants were from a convenience sample and were women only, the latter of which makes it difficult to apply the findings to men. Another limitation of the study is the low response rate (46.7%) of participants from initial recruitment to the fourth year with the consequence that the present sample was more representative of those with higher levels of eating disorder symptoms, although not general psychological distress.

Research to further investigate the findings of the present study includes more formal meditational and moderational analyses of perceived stress, psychological distress and related features such as psychological immaturity and stressful life events over time. In addition, it would be relevant to test the specific effect of interventions that aim to reducing depression and anxiety or psychological distress on eating disorder symptoms.

## Conclusions

Higher levels of perceived stress, higher levels of psychological distress, immature defense style and more frequent life events all significantly correlated with eating disorder symptoms. Psychological distress and perceived stress had the strongest independent associations with eating disorder symptoms over time.

## Abbreviations

AN: Anorexia Nervosa
BN: Bulimia Nervosa
BED: Binge Eating Disorder
EDE-Q: Eating Disorder Examination - Questionnaire
K-10: Kessler-10 Psychological Distress Scale
DSQ: Defense Style Questionnaire
PSS: Perceived Stress Scale.
